# The effect of rituximab therapy on immunoglobulin levels in patients with multisystem autoimmune disease

**DOI:** 10.1186/1471-2474-15-178

**Published:** 2014-05-25

**Authors:** Helena Marco, Rona M Smith, Rachel B Jones, Mary-Jane Guerry, Fausta Catapano, Stella Burns, Afzal N Chaudhry, Kenneth GC Smith, David RW Jayne

**Affiliations:** 1 Department of Medicine, School of Clinical Medicine, University of Cambridge, Cambridge, UK; 2 Nephrology Division, Fundació Puivert, Universitat Autònoma de Barcelona, Barcelona, Spain. Currently working on Nephrology Division, Germans Trias I Pujol, Badalona, Spain; 3 Cambridge Institute for Medical Research, Cambridge Biomedical Campus, Cambridge, UK

**Keywords:** Rituximab, Hypogammaglobulinaemia, B cell, Vasculitis, Systemic lupus erythematosus (SLE), IgG, Infection, Autoimmune

## Abstract

**Background:**

Rituximab is a B cell depleting anti-CD20 monoclonal antibody. CD20 is not expressed on mature plasma cells and accordingly rituximab does not have immediate effects on immunoglobulin levels. However, after rituximab some patients develop hypogammaglobulinaemia.

**Methods:**

We performed a single centre retrospective review of 177 patients with multisystem autoimmune disease receiving rituximab between 2002 and 2010. The incidence, severity and complications of hypogammaglobulinaemia were investigated.

**Results:**

Median rituximab dose was 6 g (1–20.2) and total follow-up was 8012 patient-months. At first rituximab, the proportion of patients with IgG <6 g/L was 13% and remained stable at 17% at 24 months and 14% at 60 months. Following rituximab, 61/177 patients (34%) had IgG <6 g/L for at least three consecutive months, of whom 7/177 (4%) had IgG <3 g/L. Low immunoglobulin levels were associated with higher glucocorticoid doses during follow up and there was a trend for median IgG levels to fall after ≥ 6 g rituximab. 45/115 (39%) with IgG ≥6 g/L versus 26/62 (42%) with IgG <6 g/L experienced severe infections (p = 0.750). 6/177 patients (3%) received intravenous immunoglobulin replacement therapy, all with IgG <5 g/L and recurrent infection.

**Conclusions:**

In multi-system autoimmune disease, prior cyclophosphamide exposure and glucocorticoid therapy but not cumulative rituximab dose was associated with an increased incidence of hypogammaglobulinaemia. Severe infections were common but were not associated with immunoglobulin levels. Repeat dose rituximab therapy appears safe with judicious monitoring.

## Background

Rituximab is increasingly used for multi-system autoimmune diseases, such as primary systemic vasculitis and systemic lupus erythematosus (SLE) [1-9]. Rituximab was licensed for the treatment of B cell lymphoma in 1997 [10], rheumatoid arthritis (RA) in 2006 [11-13] and ANCA associated vasculitis (AAV) in 2011.

Rituximab is a chimeric murine/human monoclonal antibody that results in complete peripheral blood B cell depletion for variable time periods; typically 6–12 months. During B cell depletion, new vaccine responses are impaired and a theoretical risk of new infections exists [14]. However, since CD20 is highly expressed on B cells but not on stem cells or mature plasma cells, B cell regeneration from precursors is not directly compromised by rituximab and in the short term established humoral immunity is preserved; usually with stable levels of total IgG and established vaccination antibodies [14]. However, when rituximab is used in cohorts with high prior immunosuppressive exposure, where stem cell and plasma cell compartments maybe compromised, or when rituximab is administered long term using repeat dosing strategies resulting in prolonged B cell depletion, pre-existing humoral immunity maybe impaired. Hypogammaglobulinaemia has occurred in more than 50% of non-Hodgkin’s lymphoma (NHL) patients, especially in those receiving rituximab in combination with chemotherapy or bone marrow transplantation [15-18], and less so in RA patients treated with rituximab where 3.5% had IgG levels below the normal range [19]. Hypogammaglobulinaemia has also been reported in small cohorts with primary systemic vasculitis treated with rituximab [20-23], although the impact of prior high immunosuppression exposure, and repeat rituximab dosing, as is widely used in clinical practice, are unclear.

Immunoglobulin plays a major role in adaptive immunity, and severe depletion of immunoglobulin, as observed in primary immunodeficiency syndromes increases infection risk [24]. The potential development of secondary immunodeficiency due to immunosuppressive medication, including the impact of prolonged B cell depletion on IgG levels and infection risk, is not well studied in patients with autoimmune disease.

We report on the frequency and severity of hypogammaglobulinaemia, and associated infection rates in a large cohort of rituximab treated patients with severe multi-system autoimmune diseases and prolonged follow-up.

## Methods

This was a retrospective study conducted at Addenbrooke’s Hospital, Cambridge, UK, which serves as a tertiary referral clinic and follows approximately 1000 patients with primary systemic vasculitis and SLE. In accordance with UK National Health Service Research Ethics Committee guidelines, ethical approval and patient consent were not required for this work because it comprises retrospective data and all treatment decisions were made prior to our evaluation.

All patients with multi-system autoimmune disease treated with rituximab between 2002 and 2010 were studied. 104 cases have previously been reported [3,5,25-27] and seven enrolled in a randomized controlled trial of rituximab [1] are also included in this cohort. Patients were excluded if they had fewer than six months follow-up or required repeated plasma exchange (PLEX).

### Clinical and laboratory assessments

The data was collected retrospectively from patient notes and clinical databases. Data collection included pre-rituximab demographics, disease activity assessments, medications, adverse events and immunoglobulin levels at each clinical assessment.

### Dose of rituximab

Rituximab induction therapy consisted of either 1000 mg repeated after two weeks or 375 mg/m^2^/week × 4. Patients received further rituximab at the time of relapse or at 6 monthly intervals as a remission maintenance therapy.

### Disease activity

AAV disease activity was graded by the Disease Extent Index (DEI score) [28] and by investigators’ assessment of disease activity as either full remission (DEI ≤2 and a steroid dose ≤10 mg daily), partial remission (greater than 50% reduction in DEI), ongoing disease/progressive disease (treatment failure), or relapse (increase in disease activity necessitating additional therapy beyond a temporary increase in glucocorticoid therapy).

SLE disease activity was graded by the BILAG disease activity (British Isles Lupus Assessment Group) and by investigators’ assessment of disease activity as either full remission (absence of BILAG A, B and C), partial remission (absence of BILAG A and B), ongoing disease/progressive disease (treatment failure), or relapse (increase in disease activity which required an increase in immunosuppressive therapy).

### Immunoglobulin levels

IgG hypogammaglobulinaemia was defined by serum IgG <6 g/L (normal range 6.0-13.0 g/l) for at least three consecutive months at any point during follow-up, and classified as mild (5–5.9 g/L), moderate (3–4.9 g/L) and severe (<3 g/L). IgM and IgA hypogammaglobulinaemia were defined by serum IgM levels <0.4 g/L (normal range 0.4-2.2 g/l) and IgA levels <0.8 g/L (normal range 0.8-3.7 g/l).

### Infections

Infection was defined as severe when requiring hospitalisation and/or intravenous (IV) antibiotics.

### Statistical analysis

Statistical analysis was performed using SPSS version 15 and GraphPad Prism version 5.01 (GraphPad Software, San Diego, CA). Results are expressed as values and proportions for categorical variables and medians and ranges for continuous variables. Clinical assessments and laboratory data were collected on a monthly basis. For missing values (e.g. if a patient did not attend clinic that month) a last value carried forward method was used until end of individual patient follow-up. The median interval between assessments was 3 months, with a median of 14 assessments per patient (1–55) and total patient follow-up of 8012 months. Changes in immunoglobulin levels over time were compared by a Wilcoxon signed rank test and between 2 groups by a Mann–Whitney *U* test. Proportions of patients were compared using Fisher’s exact test or Chi-squared test. Correlations were assessed using Spearman’s rank correlation coefficient. Time to first severe infection was analysed using Kaplan-Meier survival analysis with log rank analysis for significance. A family-wise p value <0.05 was considered significant for all statistical tests with appropriate adjustments being made for the multiple testing of serial data.

## Results

### Patient characteristics

One hundred and ninety-one patients received rituximab between 2002 and 2010. Fourteen were excluded; 10 due to less than six months follow-up and four due to repeated plasma exchange (PLEX). One hundred and seventy-seven patients were included (Table 1). The median age at first rituximab was 47 years (13–82); 31% were male, and the majority had primary systemic vasculitis (56%). Median disease duration before rituximab was 52 months (0–396) including 96% with relapsing/refractory disease. The median number of prior immunosuppressive or immunomodulatory agents excluding glucocorticoids was three (0–14) including prior cyclophosphamide in 121/176 (69%) with a median cumulative dose of 8 g (0–163). At time of first rituximab 72% had active disease and 28% received rituximab for persistent low grade disease activity or as remission maintenance therapy when other drugs were contraindicated. Median follow up was 43 months (2–100). All patients had at least six months of follow-up, except for four who died within six months and were included in the analysis.

**Table 1:** Characteristics and treatments of patients receiving rituximab

**Baseline Characteristics (N = 177)**	
Age (years) at first rituximab	47 (13–82)
Male sex	54 (31%)
Diagnosis	100 (56%)
Primary Systemic Vasculitis	75 (42%)
Granulomatosis with polyangiitis (Wegener’s)	15 (8%)
Microscopic Polyangiitis	10 (6%)
Churg Strauss Syndrome	43 (24%)
Systemic lupus erythematosus	3 (2%)
Behcet’s disease	3 (2%)
Henoch Schonlein Purpura	28 (16%)
Other^#^	
Prior disease duration (months)	52 (0–396)
Prior cyclophosphamide (N = 176)	121 (69%)
Cumulative cyclophosphamide (g) (N = 171)	8 (0–163)
Prior therapies (N = 176)	
Mycophenolate Mofetil	123 (70%)
Azathioprine	107 (61%)
Methotrexate	46 (26%)
Intravenous immunoglobulin	40 (23%)
Hydroxychloroquine	29 (16%)
Anti-tumor necrosis factors agents	26 (15%)
Plasma exchange	26 (15%)
Alemtuzumab	20 (11%)
Other IS/IM^$^	63 (36%)
Number of prior IS/IM agents (excluding steroids) (N = 176)	3 (0–14)
**Patient characteristics at first rituximab infusion**	
Indication	
Active disease	127 (72%)
Consolidation of remission	50 (28%)
Relapsing/refractory disease	170 (96%)
New disease	7 (4%)
Follow-up (months)	43 (2–100)
Cyclophosphamide at time of rituximab	42 (24%)
Rituximab total dose	
Total dose (g) (N = 177)	6 (1–20.2)
Dose/BSA (g/m^2^) (N = 149)	3.3 (0.8-10.4)
Dose/BSA/year (g/m^2^/year) (N = 149)	1.1 (0.1-3.2)

Data are presented for 177 patients, unless otherwise stated. Results are expressed as either medians and ranges or numbers and proportions. All values reported at/from the time of first ever rituximab.

^#^other diagnoses include neurological autoimmune disease (N = 7), cryoglobulinaemia (N = 3), urticarial vasculitis (N = 3), polyarteritis nodosa (N = 2), polychondritis (N = 2), polymyositis (N = 2), Takayasu’s disease (N = 1), membranous nephropathy (N = 1), bullous pemphigoid (N = 1) and unclassified autoimmune disease (N = 6).

^$^other IS/IM (immunosuppressant/immunomodulatory) agents include: ciclosporin (N = 15), gusperimus (N = 13), tacrolimus (N = 10), dapsone (N = 4), leflunomide (N = 4), sirolimus (N = 3), antithymocyte globulin (N = 2), colchicine (N = 2), gold (N = 2), abatacept (N = 1), mefloquine (N = 1), other (N = 6).

BSA – body surface area.

118/177 patients (67%) received 2 × 1000 mg doses of rituximab two weeks apart and 54/177 (31%), 375 mg/m^2^/week × 4. Five did not complete the induction course. 152/177 (86%) received further rituximab either for treatment of relapse or for remission maintenance. Median rituximab exposure was 6 g (1–20.2). Exposure adjusted for body surface area (BSA) was 3.3 g/m^2^ (0.8-10.4), and BSA adjusted exposure/year was 1.1 g/m^2^/year (0.1-3.2) (for the 149 patients with BSA data available). The adjustment for BSA and time was necessary as 63/177 (36%) patients received one or more BSA adjusted doses (375 mg/m^2^/week × 4) and follow-up duration was variable.

At time of first rituximab, 102/177 patients (58%) were receiving other agents; 42/177 (24%) cyclophosphamide, 28/177 (16%) mycophenolate mofetil, 10/177 (6%) hydroxychloroquine, 8/177 (5%) azathioprine, 8/177 (5%) methotrexate and 9/177 (5%) other agents. Of the 42 who received previous cyclophosphamide; 7/42 (17%) were enrolled in a randomized controlled trial (RITUXVAS) [1] and received two doses of cyclophosphamide in accordance with the trial protocol.

### Disease response

Rituximab was an effective therapy, with 151/171 patients (88%) achieving complete or partial remission by six months. Complete remission was seen in 117/171 (68%) and partial remission in 34/171 (20%). 20/171 (12%) were considered treatment failures. There was no relationship between overall response (either complete or partial remission) and the presence or absence of hypogammaglobulinaemia (IgG < 6 g/l). In addition, no relationship was identified when complete and partial remission were considered independently, or when hypogammaglobulinaemia was sub-divided into moderate-severe (IgG < 5 g/l) or mild (IgG 5–6 g/l).

### Immunoglobulin

#### Frequency and severity of low immunoglobulin levels

Immunoglobulin data was available for 136/177 patients (77%) at time of first rituximab treatment. 18/136 (13%) had IgG hypogammaglobulinaemia (5% mild, 6% moderate and 2% severe). Of the 18 that initially had IgG hypogammaglobulinaemia, this was exacerbated in 13/18 (72%) following rituximab therapy. One experienced >50% decrease in IgG level; 6 patients >25% decrease and 4 patients >10% decrease in IgG levels. At first rituximab, 14/136 (10%) had IgM hypogammaglobulinaemia and 14/136 (10%) had IgA hypogammaglobulinaemia (Table 2). Of the 118 patients who had IgG >6 g/l at time of first rituximab treatment, 27/118 (23%) subsequently went on to develop IgG hypogammaglobulinaemia, persisting for at least 3 consecutive months.

**Table 2:** Frequency and severity of low immunoglobulin levels

	**IgG**				**IgM <0.4 g/L**	**IgA <0.8 g/L**
	**< 6 g/L**	**Mild**	**Moderate**	**Severe**		
		**5-5.9 g/L**	**3-4.9 g/L**	**< 3 g/L**		
Baseline hypogammaglobulinaemia	18 (13%)	6 (5%)	9 (6%)	3 (2%)	14 (10%)	14 (10%)
Hypogammaglobulinaemia	61 (34%)	18 (10%)	36 (20%)	7 (4%)	90 (51%)	40 (23%)

Baseline hypogammaglobulinaemia (N = 136): total number and proportion in parentheses of patients with low immunoglobulin levels at time of first rituximab.

Hypogammaglobulinaemia (N = 177): total number and proportion in parentheses of patients with low immunoglobulin levels following rituximab for at least three consecutive months at some point during follow-up.

Following rituximab, 61/177 patients (34%) had IgG hypogammaglobulinaemia, for at least three consecutive months at some point during follow-up. Of these, 18/177 (10%) were mild, 36/177 (20%) moderate and 7/177 (4%) severe. After rituximab 90/177 patients (51%) had IgM <0.4 g/L and 40/177 (23%) had IgA <0.8 g/L (Table 2). During follow-up, the proportion of patients with IgG hypogammaglobulinaemia remained stable; 13% at time of first rituximab, 17% at 24 months and 14% at 60 months. Excluding those patients with hypogammaglobulinaemia at time of first rituximab, the median time to develop hypogammaglobulinaemia was 18 months (1–65), and median time to the hypogammaglobulinaemia category of maximum severity was 35 months (1–70).

#### Median immunoglobulin levels

**Figure 1:**
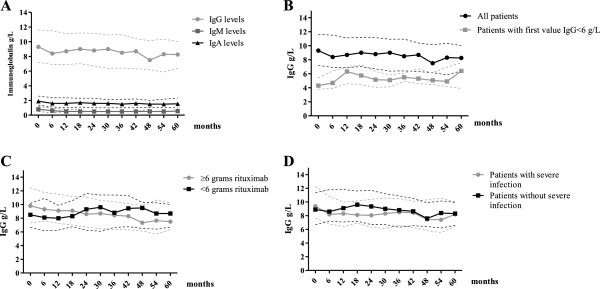
**The effect of rituximab on median immunoglobulin levels.** Median immunoglobulin levels are represented as a continuous line and interquartile ranges as dashed lines, according to time after first rituximab treatment, from 0 to 60 months. **A**. IgG, IgM and IgA levels for all 177 patients. **B**. Comparison of IgG levels for all patients with those patients with IgG <6 g/L before first rituximab (N = 18). **C**. Comparison of IgG levels for patients who received <6 g of rituximab in total (N = 79) and patients who received ≥6 g of rituximab (N = 98). **D**. Comparison of IgG levels for patients with (N = 71) and without (N = 106) severe infection.

Overall, median IgG levels remained relatively stable during follow-up, measuring at 9.3 g/L at time of first rituximab, 8.4 g/L at six months (p = 0.388) and 8.25 g/L at 60 months (N = 52) (p = 0.140). IgM levels fell from 0.8 g/L at time of first rituximab to 0.6 g/L at six months (p < 0.001) and 0.55 g/L at 60 months (p < 0.001). Baseline IgA levels were 1.9 g/L, 1.6 g/L at six months (p = 0.114) and 1.55 g/L at 60 months (p = 0.360) (Figure 1A). For those with IgG <6 g/L (N = 18) at baseline, there was a trend for levels to increase, from a median of 4.3 g/L at time of first rituximab to 4.7 g/L at six months (p = 0.276) and 6.4 g/L at 60 months (N = 7) (p = 0.099) (Figure 1B).

#### Effect of prior cyclophosphamide treatment on IgG levels

121/176 patients (69%) received prior cyclophosphamide treatment. 46/121 (38%) who received prior cyclophosphamide and 16/55 (29%) who did not, developed IgG hypogammaglobulinaemia (p = 0.308). In patients who received prior cyclophosphamide median IgG levels were lower at time of first rituximab (8.85 g/L vs. 10.4 g/L) (p = 0.025) but not at 60 months (7.9 g/L vs. 9.7 g/L) (p = 0.138) (N = 51).

#### Effect of dose of rituximab on IgG levels

39/79 (49%) who received <6 g rituximab and 32/98 (33%) who received ≥6 g rituximab developed IgG hypogammaglobulinaemia for at least 3 months (p = 0.527). Patients who received <6 g of rituximab had stable IgG levels during follow-up with a median of 8.5 g/L at time of first rituximab, 8.1 g/L at six months (p = 0.204) and 8.7 g/L at 60 months (N = 21) (p = 0.178). In patients who received ≥6 g rituximab, IgG levels trended downward from a median of 9.8 g/L at time of first rituximab to 9.3 g/L at six months (p = 0.989) and to 7.5 g/L at 60 months (N = 31) (p = 0.381) (Figure 1C).

#### Effect of glucocorticoid exposure on IgG levels

Median cumulative oral prednisolone exposure following the first rituximab infusion was 7.25 g (0–54.5). There was a negative correlation (r = −0.17 (CI −0.31 to −0.02) p = 0.02) between total oral prednisolone exposure after initial rituximab and IgG levels. Data on cumulative glucocorticoid exposure prior to rituximab therapy was unavailable. 57/177 (32%) received at least one dose of intravenous methylprednisolone following rituximab. Of those that received intravenous methylprednisolone 49% (28/57) developed hypogammaglobulinaemia compared to 28% (34/120) of those that did not (p = 0.011).

#### Effect of disease diagnosis on IgG levels

56% of patients in this cohort had primary systemic vasculitis; 24% SLE and 20% other autoimmune conditions. There was no association between diagnosis and the development of IgG hypogammaglobulinaemia (Chi-square = 3.24; df = 2; p = 0.198).

#### Severe hypogammaglobulinaemia

7/177 patients (4%) had IgG <3 g/L for at least three consecutive months during follow-up. The median age at first rituximab dose was 46 years (16–54), diagnoses were primary systemic vasculitis (N = 5), and SLE (N = 2). Median disease duration before rituximab was 57 months (8–360). The median number of immunosuppressive or immunomodulatory agents received excluding glucocorticoids was 3 [2-7] including prior cyclophosphamide in 5/7 patients (71%). Median rituximab exposure was 5.6 g (2–7.9). Two required intravenous immunoglobulin (IVIg) as replacement, 9 and 37 months after rituximab, due to recurrent infections. Four had IgG <3 g/L during the first six months, 3/4 (75%) had proteinuria ≥3 g/day with IgG levels rising with better disease control.

### Adverse events

#### Severe infections

**Figure 2:**
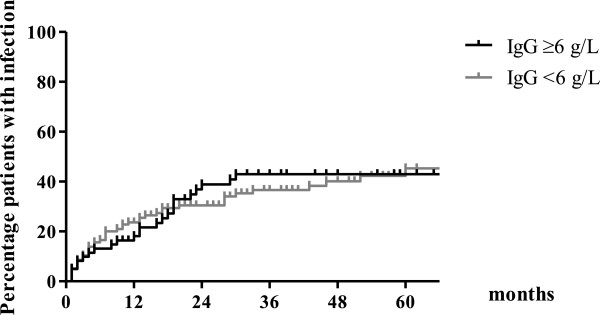
**Time to first severe infection following first rituximab.** Time to first severe infection according to IgG levels. The time to first severe infection was not different when patients with IgG levels ≥6 g/L (N = 116) and IgG levels <6 g/L (N = 61) (p = 0.953) were compared.

One hundred and seventy severe infections occurred in 71/177 patients (40%) during 8012 patient-months. Annual incidence rates of infection were 21.5 cases per 100 patient years in year 1; 10.9 in year 2; 5.2 in year 3; 1.9 in year 4 and 2.5 in year 5. The time to first severe infection was not different according to IgG levels (Figure 2).

Ninety (53%) were chest infections, 26 (15%) urinary tract infections, 12 (7%) diarrhoeal illnesses (stool culture positive), seven (4%) skin infections and 35 (21%) other infections. Seven opportunistic infections were reported in six patients; three, with granulomatosis with polyangiitis and severe cavitating lung disease had pulmonary aspergillosis (one with chronic aspergillosis diagnosed before rituximab); 2/3 patients had moderate hypogammaglobulinaemia including the patient with chronic aspergillosis who required IVIg replacement; two had infection with herpes zoster, one with moderate hypogammaglobulinaemia; and one without hypogammaglobulinaemia had both reactivation of *Cytomegalovirus (CMV)* and infection with *Mycobacterium avian intracellulari*. No cases of Hepatitis B reactivation or progressive multifocal leukoencephalopathy (PML) were reported.

45/115 patients (39%) with IgG ≥6 g/L and 26/62 (42%) with IgG <6 g/L experienced severe infections (p = 0.750) (Table 3). 43 severe infections occurred in 17/36 patients (47%) with moderate and eight in 2/7 patients (29%) with severe hypogammaglobulinaemia. There were no differences in median overall IgG levels between patients with and without infection (Figure 1D). Fourteen severe infections occurred in 7/18 (39%) with baseline IgG <6 g/L and 95 occurred in 44/118 (37%) with baseline IgG ≥6 g/L (p = 1.00). There was no difference in the proportion of patients affected by infection according IgM and IgA levels (Table 3).

**Table 3:** Severe infections according to immunoglobulin subtype and levels

	**Normal immunoglobulin levels**	**Low immunoglobulin levels**
**IgG**	**≥6 g/L**	**<6 g/L**
Patients affected	45/115 (39%)	26/62 (42%)
**IgM**	**≥0.4 g/L**	**<0.4 g/L**
Patients affected	32/87 (37%)	39/90 (43%)
**IgA**	**≥0.8 g/L**	**<0.8 g/L**
Patients affected	56/137 (41%)	15/40 (38%)

The number and proportion of patients suffering severe infections according immunoglobulin subtype (IgG, IgM and IgA) and immunoglobulin level are presented. There is no difference in the proportion of patients affected by infection when comparing IgG levels ≥6 g/L and <6 g/L (p = 0.750); IgM levels ≥0.4 g/L and <0.4 g/L (p = 0.6) and IgA levels ≥0.8 g/L and <0.8 g/L (p = 0.855).

There was a positive correlation (r = 0.265, (CI 0.118 to 0.401) (p < 0.001)) between cumulative oral prednisolone exposure and the occurrence of infection. However, no correlation was seen between risk of infection and cumulative rituximab exposure (r = −0.093 (CI −0.242 to −0.060) (p = 0.217) or prior cumulative cyclophosphamide exposure (r = 0.084 (CI −0.072 to 0.235) (p = 0.276).

#### Need for IVIg

6/177 patients (3%) received IVIg as replacement. Median age at first rituximab was 43 years (16–67); median rituximab exposure was 5.9 g (4–11.1). The median time from first rituximab treatment to start of replacement was 18.5 months [4-37]. The median IgG level before first rituximab was 4.8 g/L (4.4-5.8) and IgG level before first IVIg was 3.9 g/L (3.1-4.8). All patients had recurrent infections despite antibiotic prophylaxis and 5/6 had chronic lung disease.

#### Deaths

Thirteen patients (4 SLE, 6 primary systemic vasculitis, 3 other autoimmune diseases) died. The median age at first rituximab treatment was 62 years (43–82). Causes of death were: respiratory failure (n = 2 at 10 and 28 months after first rituximab) both with pre-existing pulmonary fibrosis; infection (n = 4 at 4, 15, 49 and 84 months) one with peritonitis, two with sepsis and active SLE with chronic kidney disease, and one with chest sepsis with chronic kidney disease and ischemic heart disease; myocardial infarction (n = 1, at 47 months), malignancy (n = 2 at 27 and 31 months); unknown cause (n = 4 at 2, 3, 5 and 16 months). 2/13 patients (15%) had mild hypogammaglobulinaemia and 3/13 (23%) moderate hypogammaglobulinaemia.

## Discussion

Rituximab is an effective therapy for remission induction in patients with multi-system autoimmune disease [1,2,26,29]. However, the majority of patients will relapse after a single course [3,5]. Further courses are effective, although concerns surround the long term effect on humoral immunity and infection risk with repeated rituximab use [30,31]. Rituximab depletes CD20+ B cells for an average of 6–12 months. Mature plasma cells (the source of 95% of circulating IgG) do not express CD20; however, prolonged depletion of plasma cell precursors may reduce replenishment of mature plasma cells leading to hypogammaglobulinaemia and infection risk.

We studied the frequency and severity of hypogammaglobulinaemia and severe infections following repeated rituximab dosing in patients with multi-system autoimmune disease. Low IgG, IgM and IgA levels were more frequent before first rituximab (13%, 10% and 10% of patients respectively) than in NHL or RA [16,17,32-34]. At 60 months, the proportion of patients with low IgG remained stable (14%) however, those with low IgM or IgA rose to 25% (p = 0.018) and 17% (p = 0.211) respectively. The increase in proportion of patients with IgM <0.4 g/L following rituximab, is consistent with previous data in SLE and AAV patients [3,20-23,26]. Low levels of IgG, IgM and IgA occurred in 34%, 51% and 23% of patients for at least 3 consecutive months at any time during follow-up. In keeping with the overall stabile proportion of patients with low IgG during follow-up, the majority of episodes of IgG hypogammaglobulinaemia were mild or transient, and only a minority suffered moderate/severe prolonged IgG hypogammaglobulinaemia. IgG levels were lower at baseline in patients who had received prior cyclophosphamide treatment. However, the development of IgG hypogammaglobulinaemia was not associated with prior cyclophosphamide exposure, but was associated with a higher cumulative corticosteroid exposure during follow-up. Median IgG levels trended downwards in those who received >6 g rituximab, although this did not reach statistical significance.

Our study is retrospective and whilst it does represent real life event rates of hypogammaglobulinaemia and severe infections, in patients with complex multisystem autoimmune disease treated with rituximab, outcomes are confounded by clinical strategies to reduce infection risk, and concomitant therapies that contribute to hypogammaglobulinaemia and infection risk. In our practice repeat rituximab dosing is used to treat and prevent disease flare irrespective of peripheral blood B cell count. Immunoglobulins are monitored at each clinic visit. Concomitant immunosuppressive therapies are generally not used alongside rituximab and during follow-up corticosteroids are reduced or withdrawn. In patients with repeated infections, antibiotic prophylaxis is employed; and in patients with infections despite antibiotics and moderate/severe hypogammaglobulinaemia (IgG < 5 g/l), IVIG replacement is used. Discontinuation of further rituximab dosing is considered in patients with IgG < 5 g/l with a downward (rather than stable) trajectory and recurrent infections; however, this decision is balanced against ongoing disease activity, the efficacy benefit derived from rituximab and the availability of other therapeutic options.

Existing data supports the safety of repeated doses of rituximab in RA and NHL, where stable infection rates were observed following initiation of rituximab treatment [33,35,36]. We observed infections in 38% of patients in the first year of treatment; similar to rates seen in other studies in primary systemic vasculitis [1,2,37,38] where active disease, chronic lung and kidney disease, prior cyclophosphamide and high corticosteroid exposure also contribute to infection risk. As in RA and NHL, the occurrence of persistent mild hypogammaglobulinaemia (IgG 5–6 g/l) following rituximab was not associated with a higher infection rate [33,35,36]. Unlike other populations, where IgG levels <5 g/l have been associated with infection risk [34], we did not observe an association; however, regular monitoring, antibiotic prophylaxis and IVIG replacement may have reduced infection rates in this subgroup.

Overall we observed a reduction in severe infection rates over time, along with improved disease control, a minimisation of corticosteroids and avoidance of concomitant immunosuppression. Our data suggest that provided careful monitoring is employed together with judicious use of prophylactic therapies, the IgM hypogammaglobulinaemia, transient mild/moderate IgG hypogammaglobulinaemia or occasional severe IgG hypogammaglobulinaemia observed with repeat rituximab dosing are not dominant factors in infection risk in a severe multisystem autoimmune disease population.

## Conclusion

We have reported on the frequency and severity of hypogammaglobulinaemia and potential infective complications following rituximab therapy in a large retrospective survey of patients with multi-system autoimmune disease with long term follow-up. Following rituximab treatment the proportion of patients with IgM and IgA hypogammaglobulinaemia rose, but the proportion with IgG hypogammaglobulinaemia remained stable. The risk of IgG hypogammaglobulinaemia was not increased by low baseline immunoglobulin levels or prior cyclophosphamide therapy or greater cumulative rituximab exposure. However, patients with high concomitant glucocorticoid doses were at risk of developing IgG hypogammaglobulinaemia.

Only a small minority developed severe IgG hypogammaglobulinaemia necessitating IVIG replacement. Severe infections were frequent in this patient population and were associated with higher glucocorticoid exposure but were not associated with IgG levels. Overall, rituximab was a safe therapy in this population. Regular immunoglobulin monitoring is indicated with repeat rituximab dosing to identify the small minority of patients with progressively falling IgG levels.

## Competing interests

Dr. Jayne has received research grant support for investigator initiated studies from Roche. Dr. Jayne and Dr. Chaudhry have received consulting fees from Roche. Dr. Jayne, Dr Jones, Dr. Smith and Dr. Chaudhry have received lecture fees from Roche. Dr. Chaudhry has received travel expenses from Roche.

## Authors’ contributions

HM, RS and RJ contributed to study design, acquisition of data, analysis of data, interpretation of the results and drafting the manuscript. MJG, FC and SB were involved in data acquisition. AC assisted with statistical analysis. DJ was involved with study design, interpretation of the results and critical review of the manuscript. KS and AC provided critical review of the manuscript. All authors read and approved the final manuscript.

## Authors’ information

HM and RS are joint first authors. They have contributed equally to the study design, acquisition of data, analysis of data, interpretation of the results and drafting the manuscript.

## Pre-publication history

The pre-publication history for this paper can be accessed here:

http://www.biomedcentral.com/1471-2474/15/178/prepub
